# The effect of a novel AQP4 facilitator, TGN-073, on glymphatic transport captured by diffusion MRI and DCE-MRI

**DOI:** 10.1371/journal.pone.0282955

**Published:** 2023-03-15

**Authors:** Alaa Alghanimy, Conor Martin, Lindsay Gallagher, William M. Holmes

**Affiliations:** 1 Institute of Neuroscience and Psychology, College of Medicine, Veterinary and Life Science, University of Glasgow, Glasgow, United Kingdom; 2 Radiological Sciences Department, College of Applied Medical Sciences, King Saud University, Riyadh, Saudi Arabia; Stanford University, UNITED STATES

## Abstract

The glymphatic system is a low resistance pathway, by which cerebrospinal fluid enters the brain parenchyma along perivascular spaces via AQP4 channels. It is hypothesised that the resulting convective flow of the interstitial fluid provides an efficient mechanism for the removal of waste toxins from the brain. Therefore, enhancing AQP4 function might protect against neurodegenerative diseases such as Alzheimer’s disease (AD), in which the accumulation of harmful proteins and solutes is a hallmark feature. Here, we test the effect of a putative AQP4 facilitator, TGN-073, on glymphatic transport in a normal rat brain by employing different MRI techniques. Surgical procedures were undertaken to catheterise the cisterna magna, thereby enabling infusion of the MRI tracer. Followed by the intraperitoneal injection of either TGN-073, or the vehicle. Using a paramagnetic contrast agent (Gd-DTPA) as the MRI tracer, dynamic 3D T1 weighted imaging of the glymphatic system was undertaken over two hours. Further, the apparent diffusion coefficient was measured in different brain regions using diffusion-weighted imaging (DWI). While physiological parameters and arterial blood gas analysis were monitored continuously. We found that rats treated with TGN-073 showed the distribution of Gd-DTPA was more extensive and parenchymal uptake was higher compared with the vehicle group. Water diffusivity was increased in the brain of TGN-073 treated group, which indicates greater water flux. Also, MRI showed the glymphatic transport and distribution in the brain is naturally heterogeneous, which is consistent with previous studies. Our results indicate that compounds such as TGN-073 can improve glymphatic function in the brain. Since glymphatic impairment due to AQP4 dysfunction is potentially associated with several neurological disorders such as AD, dementia and traumatic brain injury, enhancing AQP4 functionality might be a promising therapeutic target.

## Introduction

In humans, a quarter of the body’s total energy expenditure is consumed by the brain, and around seven grams of waste solute are excreted every day [1]. The clearance of metabolites, neurotransmitters and toxic macromolecules in an organised manner is necessary to maintain homeostasis, and prevent their accumulation and the associated initiation of pathologies. Conventionally, interstitial solutes were thought to be transported via diffusion, but recent evidence suggests an additional bulk flow of the interstitial fluid (ISF). The glymphatic (glial-lymphatic) model proposes that subarachnoid cerebrospinal fluid (CSF) is driven by arterial pulsation along the perivascular space surrounding penetrating arteries, with influx into the brain interstitium mediated by the astroglial water channel aquaporin-4 (AQP4) [2–4]. It is proposed that this influx results in a very slow bulk flow of ISF, which then exits along perivenous spaces. This bulk flow providing a more efficient clearance mechanism from the parenchyma than diffusion alone. To date, glymphatic function has been implicated in the clearance of amyloid beta, tau, alpha synuclein and lactate [2, 5–8]. In addition, it is speculated to be involved in the clearance of other solutes that cannot be locally degraded or efflux across the blood brain barrier. It has been suggested that AQP4 water channels play a crucial role by facilitating water exchange [9].

AQP water channels are plasma membrane proteins that are capable of bidirectional water movement across cell membranes. Water transported via these channels is governed by both hydraulic pressure and passive osmotic gradients. Fourteen types of AQPs have been verified as being distributed throughout the whole body of both rodents and humans. However, significant interest is directed at AQP4 due to its dense expression in the central nervous system. The distribution of AQP4 in astrocytic end feet is mainly at two regions, the perivascular space surrounding the cerebral vasculature, and the glia limitans externa underneath the pia mater [10]. Thirty-five per cent of the plasma membrane of the end feet of astrocytes is occupied by AQP4, in particular, the membrane facing the blood vasculature at the blood-brain barrier (BBB) [11, 12]. Studies using AQP4 knockout mice have shown glymphatic function is mediated by AQP4, with both the influx of CSF and efflux of ISF diminished compared to wild types [2]. Further, it has been demonstrated that the loss of perivascular AQP4 localisation impairs glymphatic function [13].

In several animal models of disease, glymphatic dysfunction has been identified. Ischaemic stroke in rodents has been associated with elevated glymphatic activity that might lead to the initiation of cerebral oedema [14]. In contrast, reduced glymphatic function has been observed in animal models of AD and traumatic brain injury [5, 15].

In-vivo human MR studies have demonstrated the presence of glymphatic function, showing brain-wide enrichment of intrathecally administered CSF tracer (Gadobutrol), with a dementia cohort exhibiting delayed tracer clearance [16]. Moreover, dynamic 11C-PiB PET has confirmed CSF-mediated clearance deficits in patients with Alzheimer disease [17, 18]. Further, post-mortem studies of Alzheimer’s patients have shown that AQP4 is upregulated [19, 20], with a loss of perivascular localisation [21]. These results indicate that altering AQP4 function pharmaceutically, by either facilitating or inhibiting water exchange, could aid in managing many neurological pathologies.

Earlier works have investigated the role of AQP4 in the brain either by inhibiting AQP4 pharmacologically or by genetically modifying rats and mice by deleting the AQP4 gene [22–24]. Using similar methods, later studies have investigated the role of AQP4 in the glymphatic system [2, 15, 25–28]. Recently, it has been shown that a readthrough extended version (AQP4X) is exclusively perivascular and that Aβ clearance is reduced in AQP4X-specific knockout mice [29]. Further, high-throughput screening has identified small molecule compounds that enhance readthrough of the AQP4 sequence [29].

In addition, a novel AQP4 facilitator (TGN-073) has been identified [10]. It has been postulated that ligand interaction with AQP-4 leads to a conformational shift, especially in the protein loop spanning the H2 and HB helices increasing the channel diameter and increasing water flux [30]. However, it is also possible that TGN-073 exerts an indirect effect on AQP4. For example, by impacting translational readthrough of AQP4 [29], by impacting subcellular localisation of AQP4 [31] or via some other unknown mechanism. Here we seek to investigate the effect of this novel AQP4 facilitator on the glymphatic transport in the normal rat brains using dynamic contrast-enhanced-MRI (DCE-MRI) and assess changes in tissue water diffusivity using Diffusion MRI.

## Methods and materials

### Animals

Male Wistar rats were employed in the experiment (Charles River; aged: 20 to 24-weeks old, body weight: 300–380 g). Animal groups: vehicle (n = 7), negative control aCSF (n = 3) and TGN-073 treated group (n = 7). Rats were randomly assigned to either the treated or the vehicle group. Two rats were excluded from the study, one vehicle and one TGN-073 treated, due to sudden death and unstable blood pressure, respectively. All animals were housed in the MRI unit one week prior the study for acclimatisation to reduce variabilities in results. Food and water access was *ad libitum*, and 12 h dark and 12 h light cycle was maintained. Humidity (53% ± 2%), ventilation and temperature (21.5°C ± 0.5°C) were controlled automatically. The sample size was based on literature [32].

Heart rate, respiration rate and blood pressure were monitored throughout the experiments along with arterial blood gases. The rats’ mean physiological signs were: respiration 65 ± 5 breaths per minute, heartbeats per minute 400 ± 50 and mean blood pressure 95 ± 5 mmHg. In addition, the core body temperature was monitored throughout the whole experiment and was maintained at 37.0 ± 0.5°C using a rectal thermocouple and controlled by a heating pad (Polystat^®^ Cole-Parmer). At the end of scanning, animals were euthanised.

### Drug preparation

The AQP4 facilitator, TGN-073, N-[3-(benzyloxy)pyridin-2-yl] benzenesulfonamide (Key Organics, Ltd, Camelford, Cornwall, UK) was purchased in bulk. Gamma-cyclodextrin solution (10 mM) (Sigma-Aldrich, St. Louis, Missouri, USA) and 5% DMSO; 250 μl of DMSO and 4750 μl of sterile H_2_O (DMSO, Sigma-Aldrich, 10 mM) were used to increase the solubility of TGN-073 [33]. The drug was added to the suspension, along with a magnet. The sealed container was placed on the magnetic stirrer at 500rpm and stirred for 30 min before injection. In the TGN-073 treatment group, the drug was injected intraperitoneally using a 25G needle (200 mg/kg in 20ml/kg body weight). Meanwhile, the vehicle group only received gamma-cyclodextrin and DMSO dissolved in distilled water, 30 min prior to starting the MRI study.

### Animal surgery

**Fig 1:**
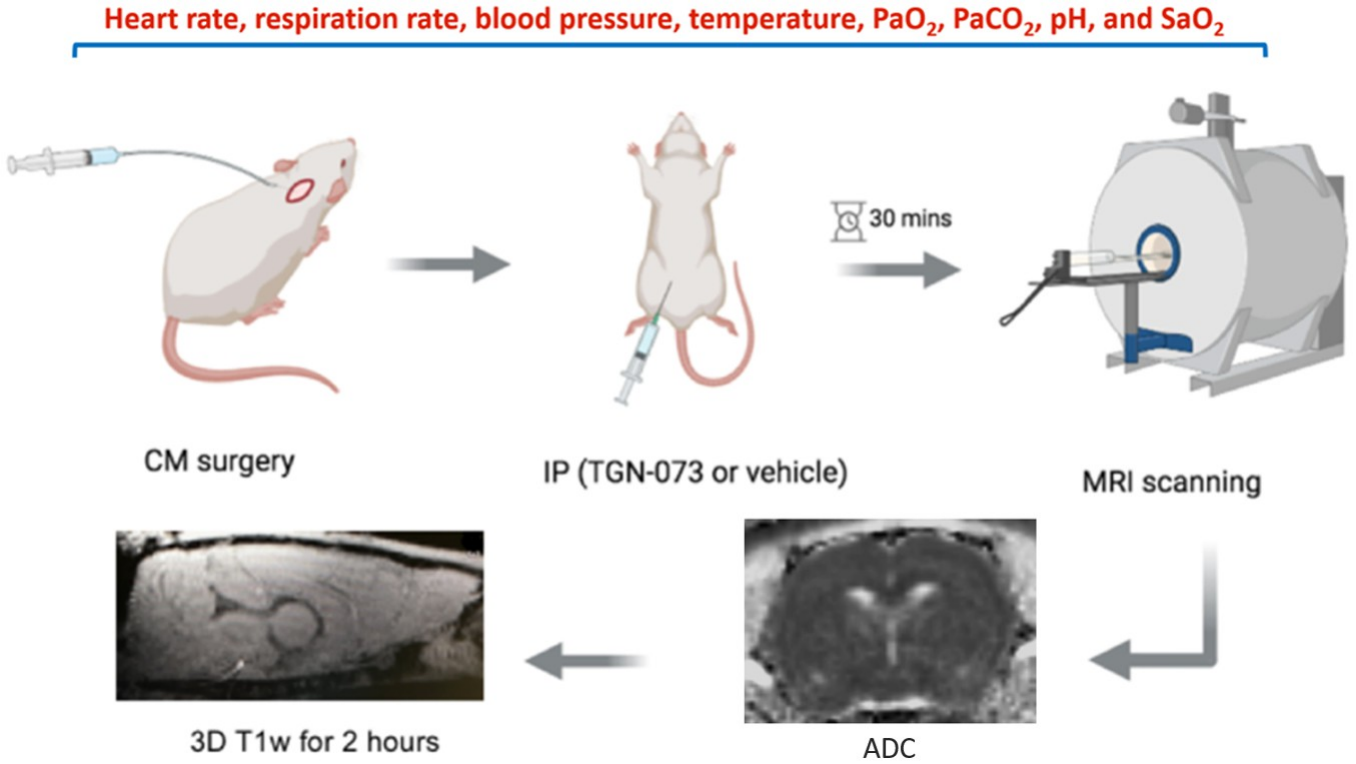
Study design (protocol). Cisterna magna cannulation then intraperitoneal injection of the drug; wait half an hour and start MRI scanning. CM: cisterna magna, IP: intraperitoneal, ADC: apparent diffusion coefficient, and MRI: magnetic resonance imaging.

The study design is shown in Fig 1. The anaesthetic regimen was as follows: initially, all rats were induced with 5% isoflurane inside an induction chamber using O_2_ and N_2_O as carrier gases (0.3 and 0.7 l/min respectively). Then, when the rats lost consciousness (i.e. did not respond to toe pinch) the animal was transferred onto facemask and isoflurane was reduced to 1.5%–2.5%, while keeping the same volume of O_2_ and N_2_O (ratio 30:70). Next the surgical areas were shaved and sterilised. The animal was then intubated via tracheotomy for artificial ventilation using a 13G feeding tube and connected to a ventilator (Harvard Apparatus). A homeothermic blanket system with a rectal probe was used. Cannulation of the femoral artery was performed with polythene tubing PE-50 (diameters: 0.58 mm ID, 0.96 mm OD, Smith Medical International Ltd) and this was connected to a blood pressure transducer to measure arterial BP and HR continuously (Biopac Systems, MP100), and for the regular analysis of arterial blood gases and pH (epoc^®^ Blood Analysis System, Siemens Healthineers). Parameters were maintained as follows: partial pressure of oxygen 90-110mmHg, partial pressure of carbon dioxide 35-45mmHg, pH 7.4 ± 0.1, and O_2_ saturation: 97%–100%.

**Fig 2:**
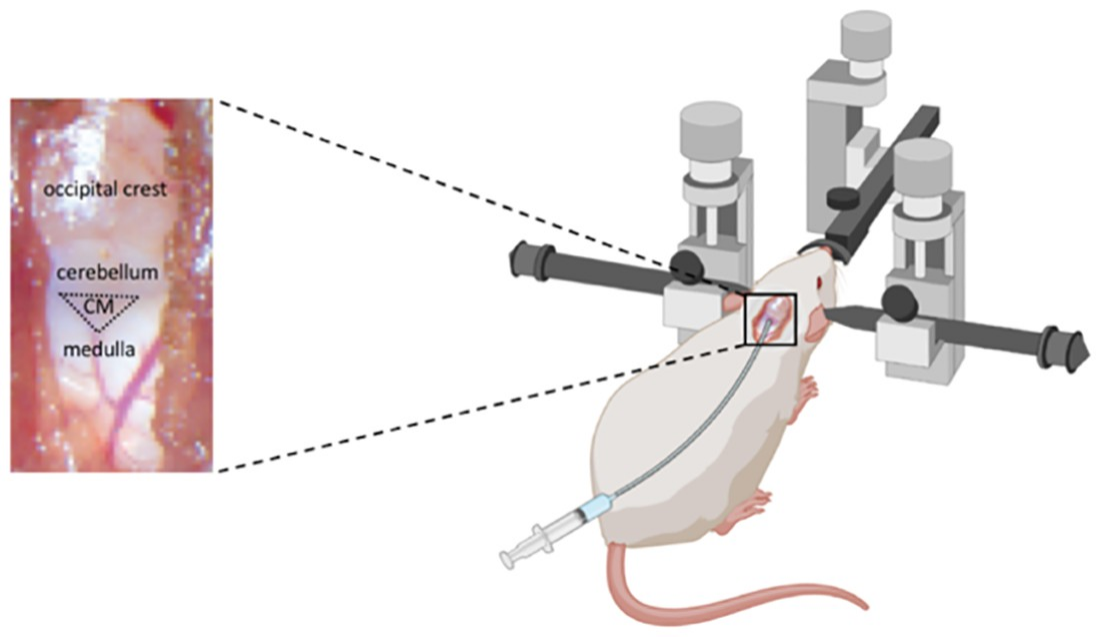
Cisterna magna cannulation. Rat was placed in the prone position in a stereotaxic frame, using tooth and ear bars for stabilisation with the head fixed to 45° (snout). A 3 cm skin incision was made in the midline of the dorsal neck to expose the occipital crest and dural membrane covering the cisterna magna. A custom-made CM cannula (22-gauge, 2 mm Tip PEEK, SAI Infusion Technologies, RCMC-03) was advanced 2 mm into the subarachnoid space (i.e. CM space). CM: cisterna magna.

Each rat was placed in the prone position in a stereotaxic frame, using tooth and ear bars for stabilisation with the head fixed to 45° (snout), Fig 2. After sterilising the area, to access the cisterna magna (CM), a 3 cm skin incision was made in the midline of the dorsal neck to expose the occipital crest and dural membrane covering the cisterna magna. The muscles were separated to expose the atlanto-occipital membrane that overlays the dura mater. A custom-made CM cannula (22-gauge, 2 mm Tip PEEK, SAI Infusion Technologies, RCMC-03) was connected to a polyethylene tube and filled with artificial CSF (aCSF: NaCl 140 mmol/L, NaH_2_PO_4_ 12 mmol/L, KCl 3 mmol/L, CaCl_2_ 2.5 mmol/L, NaHCO_3_ 12 mmol/L, pH 7.4) then advanced 2 mm into the subarachnoid space (i.e. CM space), Fig 2. The cannula was secured in place with super glue to avoid any movement or leakage. Then, the muscles and overlying skin were sutured to close the skull. Next, the animal was placed in the prone position in the cradle and moved to the MRI scanning room. The animal’s physiology and vital signs were monitored continuously during the scanning with an MRI-compatible assembly.

### Infusion pump setup

In Wistar rats the intracranial pressure (ICP) starts to increase when the intracisternal infusion rate is above 3 μl/min [34]. Previously, we have performed intracisternal infusion of Gd-DTPA at 1.8 μl/min [35]. However, in pilot experiments this rate induced unstable blood pressure in this batch of Wistar rats. Reducing the infusion rate to 1.6 μl/min, as originally used by Iliff [32], resulted in stable blood pressure.

The polyethylene tube (Smith Medical International Ltd) was filled with MRI contrast (Gd-DTPA) or artificial CSF (aCSF) and connected at one end of the CM cannula and the distal end was connected to a long line extending from the microinfusing pump (Carnegie Medicie CMA|100 Microinjection Pump with CMA|111 Syringe Selector). The accuracy of the infusion pump was assessed by setting a specific flow rate and time period and weighing the amount of solution delivered. A total of 50 μl of the Gd-DTPA or aCSF was delivered intracisternally at an infusion rate of 1.6 μl per minute with total infusion time = 31 minutes.

### Magnetic resonance imaging

All imaging was performed on a 7Tesla preclinical MRI system (Bruker PharmaScan 7T/16) controlled by Paravision 5.1 software (Bruker Bio Spin, Ettlingen, Germany) and gradient coils with 9 cm inner diameter and a maximum strength of 300 mT/m. A surface coil with a four-channel phased array was used as the RF receiver coil and a volume (birdcage) coil with 72 mm inner diameter was used as RF transmitter. Ear and mouth bars were used for head stabilisation.

The scanning protocol was as follows: first, a localiser scan was performed to adjust the slice orientation and position. Second, a 3D T1-weighted fast low angle shot (FLASH) sequence was used to image the transport of Gd-DTPA. The scan parameters were as follow: TR = 15 ms, TE = 3.1 ms, flip angle = 15°, number of averages = 1, field of view = 3.0 × 3.0 × 3.0 cm, acquisition matrix size of 128 × 128 × 128 yielding an image resolution of 0. 234 × 0.234 × 0.234 mm/pixel, acquisition time = 2 min 55 s, acquired in the sagittal plane. The scan protocol included five 3D T1 weighted FLASH baseline scans (five baseline images were obtained to measure the noise in relation to the true signal change induced by CSF tracer infusion). This was followed by the intrathecal infusion of paramagnetic contrast agent through the cannula in the CM; either Gd-DTPA (21 mM, molecular weight 938 Da, Magnevist^®^ Bayer HealthCare Pharmaceuticals, Inc.) or aCSF was used as a negative control, during the scan. The total volume of Gd-DTPA or aCSF administered was 50 μl at an infusion rate of 1.6 μl per minute; total infusion time = 31 minutes. After finishing the contrast infusion, the MRI scanning acquisitions continued for a total scanning time of two hours from the beginning of MRI contrast infusion.

To confirm the effect of TGN-073 on the astrocytic microstructure, diffusion-weighted echo planar imaging (DW-EPI) spin echo sequences were performed before contrast agent administration. The scan parameters were TR = 4000 ms, TE = 23.2 ms, b-values (0, 1000) s/mm^2^ along three orthogonal axes, diffusion time = 30 ms, NA = 2, FOV = 2.50 x 2.50 cm, acquisition matrix = 96, slice thickness = 1.50 mm, number of contiguous slices = 8, acquisition time = 2 min 8 s, acquired in the coronal plane. At the end of the study, the animal was euthanised with an overdose of isoflurane.

### Data analysis

To avoid bias, the coding of rats and data analysis were carried out in a blind fashion (i.e. the investigator was not aware whether rat received the drug or vehicle). All image data processing was performed using MATLAB software (MATLAB R2019a, MathWorks Ltd., UK); codes were developed in house. In brief, the DICOM format was used to process the MRI images. Brain extraction was achieved by applying a mask to get rid of non-brain tissue, which allowed for accurate brain co-registration. For spatial alignment, images were co-registered with rigid body transformation to the baseline image to correct for any inter-imaging head movement. Then, image smoothing was achieved by applying anisotropic diffusion filters to reduce noise.

A visual inspection was carried out to assure adequate pre-processing. Next, the averaged baseline image was subtracted from all of the time series images (i.e. after initiation of the contrast infusion). Then, subtracted images were divided by average baseline (image normalisation); this was carried out to correct for any signal variations due to nonuniform sensitivity of the surface RF receiver coil. The resulting value was then multiplied by 100 to find the percent change in signal intensity. Mean percentage signal intensity changes were extracted from regions of interest (ROI) drawn within the frontal cerebral cortex, cerebellum and whole brain. Plotting these percentage signal changes as a function of time, gave the time activity curves (TAC) for each region.

The DWI-EPI images were analysed using image processing tools supplied by Bruker Paravision 5.1 software. Two animals (a drug treated and a vehicle) were excluded from the DWI calculations due to EPI artefacts in the Z-direction. Total animal numbers used for the DWI analysis were n = 6 drug, and n = 6 vehicle. The diffusion coefficient (D) was calculated:

$$\text{D}=-\text{ln}\left({\text{S}}_{\text{b}1000}/{\text{S}}_{\text{b}0}\right)/\text{b},$$ (1)

The apparent diffusion coefficient (ADC) was calculated from the average of three orthogonal directions:

$$\text{ADC}=\left(\text{Dx}+\text{Dy}+\text{Dz}\right)/3$$ (2)

where b is the b value, S_b0_ is the signal intensity with zero diffusion gradients (b = 0), S_b1000_ is the signal intensity from the trace-DW image, Dx is the diffusion coefficient in the x direction, Dy is the diffusion coefficient in the y direction, and Dz is the diffusion coefficient in the z direction. For each rat, the cerebral cortex, striatum and whole brain were manually segmented in the ADC map to find the average apparent diffusion coefficient values.

### Statistical analyses

Statistical analyses and graphs were plotted using MATLAB software (MATLAB R2019a, MathWorks Ltd., UK), codes developed in house, Excel 2016 (Microsoft Windows), and GraphPad Prism 9 (GraphPad Prism Software, California, USA). Statistical comparisons between the drug and vehicle groups were performed by repeated measures one-way ANOVA followed by Tukey’s post hoc test to correct for multiple comparisons. A two-tailed unpaired (independent groups) student t-test was performed to study the ADC difference between the drug and vehicle groups. Data are presented here as mean ± SD unless otherwise stated, with P<0.05 considered to be statistically significant.

### Study approval

All animal imaging and surgical procedures were approved by the University of Glasgow committee and the UK Home Office. All experiments were performed following the regulations of the ASPA (Animals Scientific Procedures Act 1986) and the European legislation for animal protection, Directive 2010/63/ EU.

## Results

**Fig 3:**
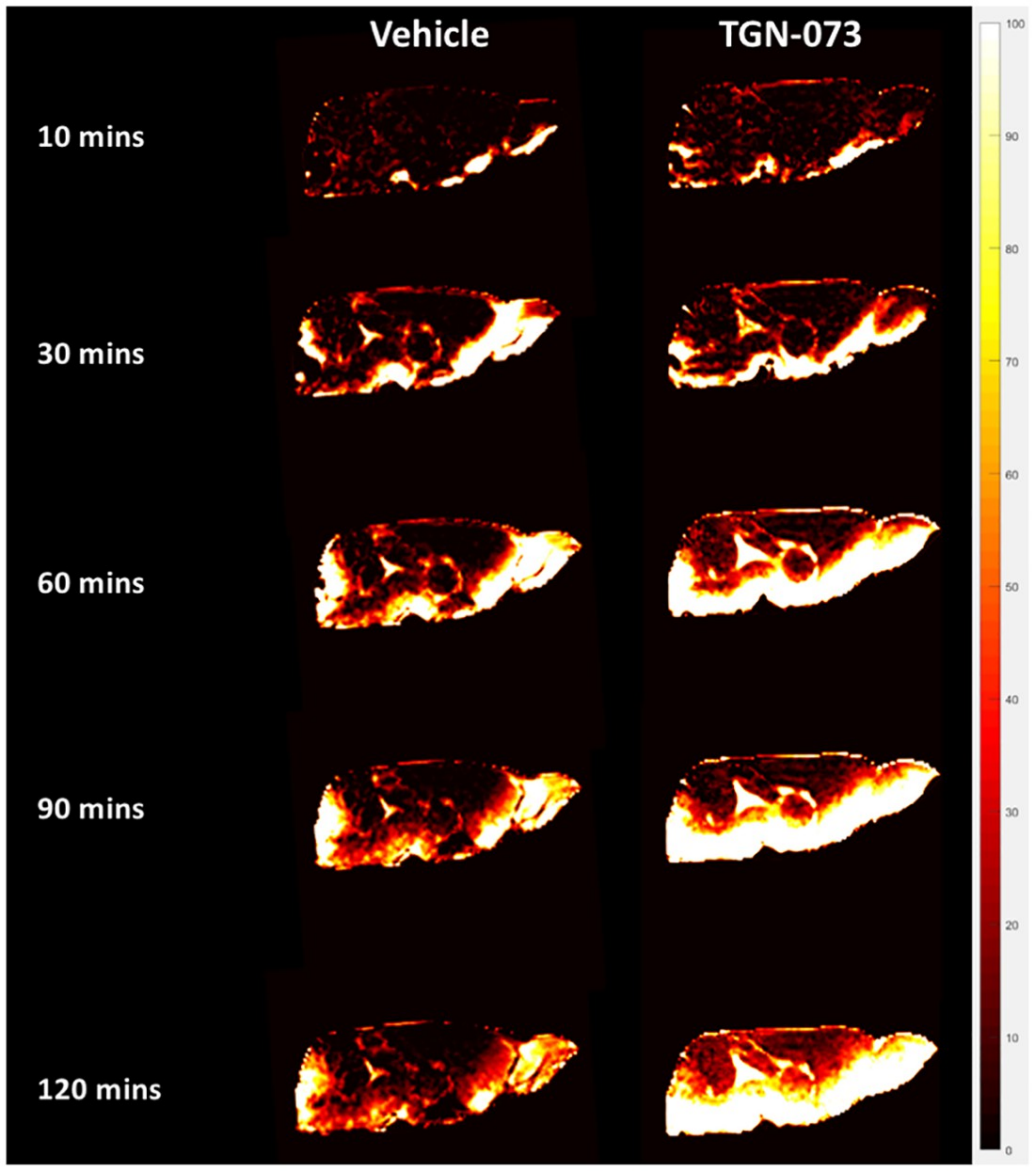
Serial MRI images in the sagittal plane of a rat’s brain. Using Gd-DTPA as the paramagnetic contrast agent reveals glymphatic transport with higher uptake and more parenchymal penetration over the whole brain over a period of two hours in the animals treated with the AQP4 facilitator (TGN-073) compared to vehicle-only treated rats. The time (in minutes) shows the change over time from starting the Gd-DTPA infusion.

**Fig 4:**
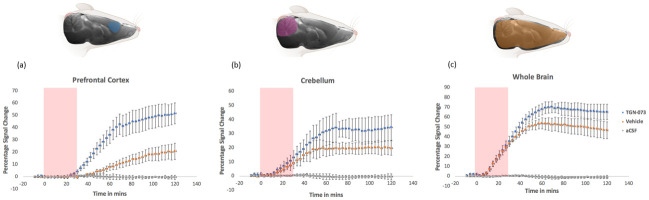
Averaged time activity curves (TAC) of three different regions of rats’ brains. The percentage signal change is plotted as a function of time for theTGN-073 treated (n = 6, blue circles), vehicles (n = 6, orange circles), and artificial CSF (n = 3, grey circles) in (a) prefrontal cortex, (b) cerebellum and (c) whole brain. Pink shading on graphs indicates the period of tracer infusion. Shading on the anatomical images of the brain illustrates the location of ROIs, blue: prefrontal cortex, purple: cerebellum and orange: whole brain.

Direct imaging of the effect of the AQP4 facilitator, TGN-073, upon the glymphatic transport in rat brain was accomplished via intrathecal infusion of paramagnetic contrast agent Gd-DTPA. The images from serial 3D T1-weighted MRI clearly show more contrast uptake and deeper tissue penetration in the brain of the drug group compared to the vehicle group (Fig 3). This effect was markedly noticeable in the TAC of the prefrontal cortex, cerebellum, and whole brain (Fig 4a–4c). The prefrontal cortex showed more than twofold higher contrast uptake in the treated group than the vehicle group (Fig 4a). The whole brain TAC of the TGN-073 treated animals revealed an increase of up to 41% in contrast enhancement compared to that of the vehicle group (Fig 4c). While the cerebellum showed higher difference between the groups, with a 70% increase in contrast enhancement in the treated group compared to the vehicle group (Fig 4b).

In the prefrontal cortex, the contrast uptake was substantially greater in the TGN-073 group compared to vehicle (51% ± 8% vs 20% ± 6%, respectively; P = 0.0001). In the cerebellum, the contrast uptake was also substantially greater in the TGN-073 group compared to vehicle (34% ± 9% vs 20% ± 5%, respectively; P = 0.0004). For both the vehicle and TGN-073 treated groups, in the cerebellum the time activity curves (TAC) showed a plateau after approximately one hour (Fig 4b). However, in the prefrontal cortex the TAC continued to increase right to the end of the 2 hour scanning period (Fig 4a).

The glymphatic transport and distribution in the brain is naturally heterogeneous (Fig 3). For instance, in TGN-073 treated group, T1-weighted MRI dynamic time series show heterogeneity in the arrival time of MRI tracer and maximum signal change in different brain regions. For the arrival time of the contrast in the TGN-073 group in TAC (Fig 4), the cerebral cortex signal started to increase after 30 min of starting the contrast infusion; meanwhile, the cerebellum signal started to increase just after 12 min of starting the infusion. The maximum percentage signal change was higher in the cerebral cortex than the cerebellum (51% ± 8% vs 34% ± 9%, respectively; P < 0.001). Thus, glymphatic transport differs in different brain territories.

**Fig 5:**
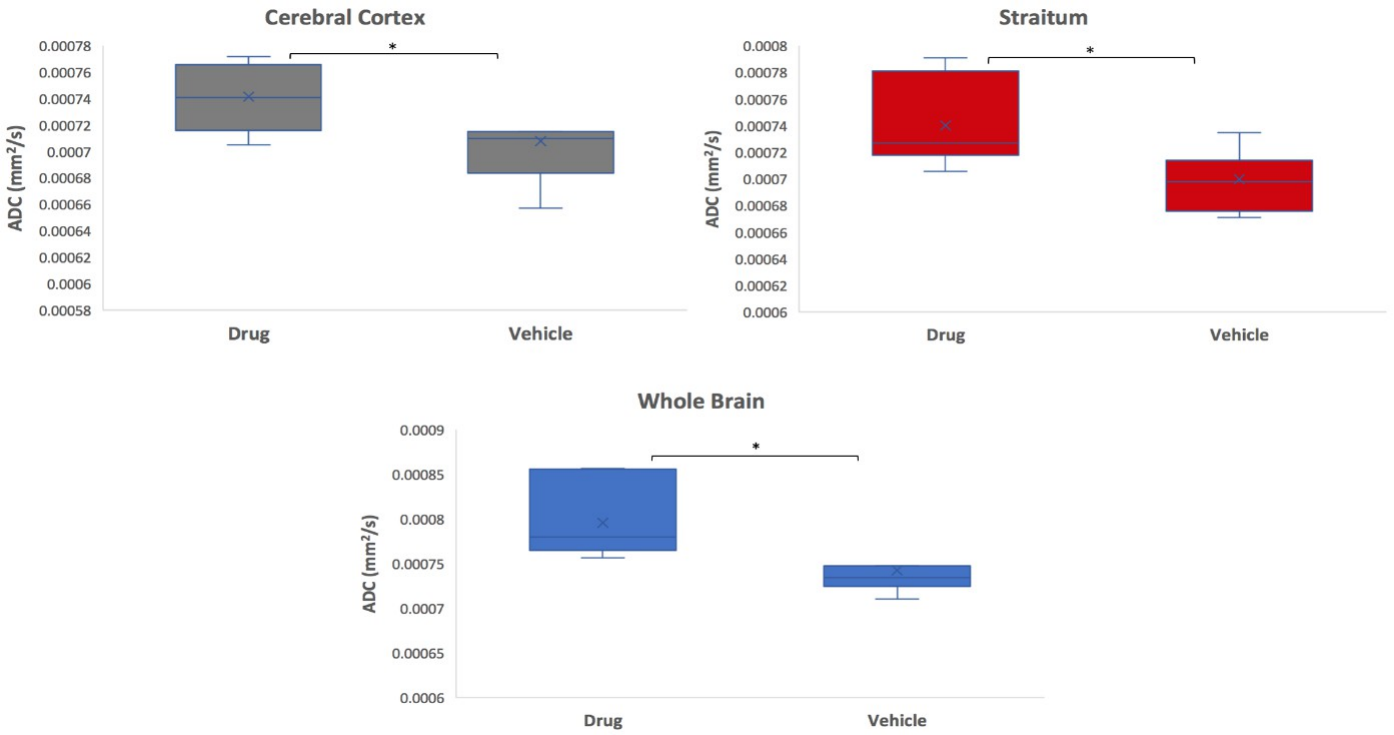
Boxplots of apparent diffusion coefficient in the cerebral cortex (grey), the striatum (red), and whole brain (blue). In each figure, left: TGN-073-treated group; right: vehicle group. Asterisks indicate significant differences: *P < 0.05.

Rats treated with TGN-073 showed increased water diffusion than those treated with vehicles (Fig 5). The boxplot of different ROIs in the brain reveals that ADC was higher in the TGN-073 treated group than in the vehicle group (Fig 5). Significant differences in ADC values were observed in the cerebral cortex (0.00074 vs 0.00070 mm^2^/s; P < 0.05), striatum (0.00074 vs 0.00069 mm^2^/s; P < 0.05), and whole brain (0.00079 vs 0.00074 mm^2^/s; P < 0.05).

### Supplemental video

The supplemental video shows side-by-side the temporal development of contrast agent (Gd-DTPA) in the brain of TGN-073-treated and vehicle rats over two hours (S1 Movie).

## Discussion

Reduced glymphatic activity is associated with several neurological disorders such as AD, traumatic brain injury and vascular dementia [5, 15, 36]. In Alzheimer’s disease, it is speculated that the accumulation of harmful proteins (Amyloid β and tau) is the result of glymphatic dysfunction. For example, experiments have shown that injecting the striatum of AQP4-/- mice with ¹²⁵I-Aβ₁–₄₀ results in a 55% inhibition in the clearance of Aβ compared to their wild-type counterparts [2]. Moreover, a 40% reduction in the clearance of ¹²⁵I-Aβ₁–₄₀ has been shown in the brain of aged mice, possibly due to reduced arterial pulsatility, along with changes in AQP4 expression [37]. Therefore, pharmacological interventions that enhance water exchange through AQP4 and enhance clearance via glymphatic transport might be promising therapeutics for neurodegenerative diseases, by preventing the accumulation of harmful solutes in the brain. A previous study using intravenous injection of H_2_^17^O has demonstrated that TGN-073 facilitates the transport of water through AQP4 channels across the blood brain barrier [10]. In this study, we sought to investigate the effect of TGN-073 on glymphatic transport.

The contribution of astrocytic water channel AQP4 to glymphatic transport is under considerable debate [38–42]. This study was conducted to investigate the role of AQP4 by employing the novel AQP4 facilitator TGN-073 in normal rat brains.

Glymphatic transport was imaged using DCE-MRI with Gd-DTPA, where the contrast agent was slowly infused into the CSF at the cisterna magna. In the vehicle group the parenchymal uptake of Gd-DTPA shows a similar temporal distribution to that seen in previous studies [32, 35]. In the cerebellum the time activity curves (TAC) show a plateau after approximately one hour (Fig 4b). This can be explained by two key features. First, the cerebellum is adjacent to the infusion site of the cisterna magna, while it takes much longer for the contrast agent to reach more distal sites, e.g. the prefrontal cortex. Second, the high expression of AQP4 in the cerebellum [43, 44], could enable faster uptake and transport, reaching saturation sooner. Whereas, in the prefrontal cortex the TAC continued to increase right to the end of the 2 hour scanning period (Fig 4a).

We found that glymphatic transport was significantly enhanced in TGN-073-treated rats compared to vehicles (Fig 3). The brain of the treated group showed higher percentage signal change and deeper penetration of the contrast agent than the vehicle group. It has previously been reported that entry of tracers from the peri-arterial space to the surrounding brain interstitium was restricted based on molecular weight [32]. Very large molecular weight tracers, like FITC-d2000 (MW 2,000 kDa), were confined to the peri-arterial spaces, but lower molecular weight tracers, including Texas Red-conjugated dextran (MW 3 kDa) and Alexa Fluor 647-conjugated ovalbumin (MW 45 kDa), quickly penetrated into the interstitium [2]. Given that the size of the narrowest part of the pore of AQP4 channel is around 2.8 Å (angstroms), just large enough to let water molecules pass through in a single file [45–47], it is clear that Gd-DTPA (938 Da) cannot pass through AQP4 channels. However, Gd-DTPA can penetrate the 20 nm cleft between overlapping astrocytic end feet, which entirely surround the cerebral vasculature [48].

Given the above consideration, it is worth questioning how an AQP4 facilitator, e.g. TGN-073, could increase the rate at which Gd-DTPA penetrates the brain. We speculate that TGN-073 facilitates faster transport of water from the periarterial space into the brain interstitium, by lowering the resistance to flow. As a consequence, we posit that water flow (i.e. CSF flow) in the periarterial space is increased. As Gd-DTPA is dissolved in the CSF, and is transported with it, this in turn increases the rate at which Gd-DTPA can enter the interstitium.

Diffusion MRI is extremely sensitive to alterations in tissue microstructure and does not require any contrast agent. A number of studies have used diffusion MRI to investigate glymphatic transport [49–52]. In addition, diffusion MRI has been used to study AQP expression and its relationship to several diseases [53]. Mukherjee et al. showed that AQP1 overexpression correlated with higher water diffusivity [54]. It was shown that the severity of hydrocephalus correlates with the ADC and AQP4 expression [55]. Meanwhile, TBI rat models showed decreased water diffusivity after inducing small interfering RNAs to inhibit AQP4 [56]. Another study documented the reduction in water diffusivity in rodents’ brains, and ADC values were reduced by 50% after acutely inhibiting AQP4 using RNA interference [24]. In line with these studies, our results show a significant increase in ADC values in the group given the AQP4 facilitator TGN-073. This may indicate that by increasing the flux of water through AQP4 channels, TGN-073 reduces restrictions to the diffusion of water molecules in the brain parenchyma. However, it is also possible that this increase in ADC is the result of increased brain water content rather than changes in water transport via AQP4. Previous studies have related ADC changes to changes in brain water content measured by wet/dry weights [57, 58]. However, in our case, given the relatively small changes in ADC (~6%) seen with TGN-073 and the variability of wet/dry measurements, it would be difficult to conclusively determine whether brain water content changes or not.

## Conclusion

The glymphatic system is a low resistance pathway, in which cerebrospinal fluid enters the brain parenchyma along perivascular spaces via AQP4 channels. It has been hypothesised that the resulting convective transport of the interstitial fluid provides an efficient mechanism for the removal of waste toxins from the brain; this process is thought to be highly mediated by AQP4 water channels. Therefore, maintaining and supporting AQP4 function might protect against neurodegenerative diseases such as Alzheimer’s disease. Here, we used DCE-MRI to demonstrate that glymphatic transport is enhanced by an AQP4 facilitator, TGN-073. Further, diffusion MRI measurements demonstrated an increase in the diffusive transport of water in the brain of TGN-073 treated rats. Drugs like this novel AQP4 facilitator might hold a promising future in preventing, treating, or ameliorating neurodegenerative diseases in which the AQP4 functionality is impaired.

## Supporting information

S1 Movie The temporal development of contrast agent (Gd-DTPA) in the brain of TGN-073-treated and vehicle rats. (MP4)

S1 File Diffusion weighted imaging dataset. (ZIP)

S2 File T1 FLASH MRI dataset. (ZIP)
